# Evaluation of anti-smoking television advertising on tobacco control among urban community population in Chongqing, China

**DOI:** 10.1186/s12971-015-0057-4

**Published:** 2015-09-04

**Authors:** Xianglong Xu, Tao Gong, Yong Zhang, Chengbin Wu, Yao Jie Xie, Harry HX Wang, Runzhi Zhu, Wentao Li, Libin An, Yong Zhao

**Affiliations:** School of Public Health and Management, Chongqing Medical University, No. 1, Yixueyuan Road, Yuanjiagang, Chongqing, 400016 China; Research Center for Medicine and Social Development, Chongqing Medical University, Chongqing, 400016 China; The Innovation Center for Social Risk Governance in Health, Chongqing Medical University, Chongqing, 400016 China; Chongqing Health Education Institute, Chongqing, China; School of Nursing, The Hong Kong Polytechnic University, Hong Kong, SAR Hong Kong; School of Public Health, Sun Yat-Sen University, Guangzhou, 510080 P.R. China; General Practice and Primary Care, Institute of Health and Wellbeing, University of Glasgow, Glasgow, G12 9LX UK; School of Nursing, Dalian University, Liaoning, China

**Keywords:** Anti-smoking, television advertising, evaluation, urban community, China

## Abstract

**Background:**

China is the largest producer and consumer of tobacco in the world. Considering the constantly growing urban proportion, persuasive tobacco control measures are important in urban communities. Television, as one of the most pervasive mass media, can be used for this purpose.

**Methods:**

The anti-smoking advertisement was carried out in five different time slots per day from 15 May to 15 June in 2011 across 12 channels of Chongqing TV. A cross-sectional study was conducted in the main municipal areas of Chongqing. A questionnaire was administered in late June to 1,342 native residents aged 18–45, who were selected via street intercept survey.

**Results:**

Respondents who recognized the advertisement (32.77 %) were more likely to know or believe that smoking cigarettes caused impotence than those who did not recognize the advertisement (26.11 %). According to 25.5 % of smokers, the anti-smoking TV advertising made them consider quitting smoking. However, females (51.7 %) were less likely to be affected by the advertisement to stop and think about quitting smoking compared to males (65.6 %) (OR = 0.517, 95 % CI [0.281–0.950]). In addition, respondents aged 26–35 years (67.4 %) were more likely to try to persuade others to quit smoking than those aged 18–25 years (36.3 %) (OR = 0.457, 95 % CI [0.215–0.974]). Furthermore, non-smokers (87.4 %) were more likely to find the advertisement relevant than smokers (74.8 %) (OR = 2.34, 95 % CI [1.19–4.61]).

**Conclusions:**

This study showed that this advertisement did not show significant differences on smoking-related knowledge and attitude between non-smokers who had seen the ad and those who had not. Thus, this form may not be the right tool to facilitate change in non-smokers. The ad should instead be focused on the smoking population. Gender, smoking status, and age influenced the effect of anti-smoking TV advertising on the general population in China.

## Background

Approximately 28.1 % persons (301 million) aged 15 years and above are smokers in China [1]. Of non-smokers, 72.4 % claim they had been exposed to secondhand smoke and 38.0 % report being exposed to secondhand smoke daily [1]. Worldwide, the leading causes of premature death are smoking-related diseases, such as cancer, and cardiovascular diseases [2]. China is facing a growing burden of chronic diseases and unhealthy lifestyles. In particular, smoking is a major independent predictor for disease and subsequent health service utilization [3, 4].

Mass media communication is critical in advocating for implementing and enforcing smoke-free policies. Mass media campaigns (MMCs) have been implemented for tobacco control in several cities in China, such as Beijing, Guangdong, and Chongqing, since the 1970s. Two 30-s ads were developed to convey the message that giving the gift of cigarettes equals giving the gift of illness and death. The ads were broadcasted on regional cable TV networks from December 2008 to February 2009 and on one national satellite channel in Guangdong province [5]. Considering that China is a developing country, television is the generally accepted entertainment medium and is the most dominant medium in China. The number of subscribers of Cable TV reached 187.3 million and TV integrated coverage of the Chinese population reached 97.62 % as of 2010 [6]. However, very few anti-tobacco TV advertisements have aired in recent years despite the massive efforts of the Chinese government toward tobacco control. Studies have shown that tobacco-control mass media campaigns could encourage people to refrain from smoking [7]. Previous research has shown that mass media anti-smoking campaign prompts help-seeking behaviors among individuals exposed to the advertisement, such as calling the smoking cessation hotline [8–10], increased quitting intentions, increased cessation activity, and reduced smoking rates. MMCs increased the likelihood of tobacco control policy in prompting smokers to attempt quitting [11]. With support from tobacco control policy enforcement, campaigns could have affected larger population coverage. Considering that China is the largest country that produces and consumes tobacco, the implementation of anti-smoking advertising is imperative. However, the effectiveness of the policy is dependent on the type and the intensity of the campaign [12]. Current evaluation of the effectiveness of anti-tobacco MMCs mainly concentrates on the total population [13]. Few studies have examined the memory recall and subsequent evaluation of anti-tobacco TV advertisements by the urban community population. Moreover, Western China lacks research evaluating the effectiveness of anti-smoking TV advertising.

The Chongqing Smoking and Health Association commissioned an advertisement entitled “An Invisible Killer in the Office,” which was broadcasted daily from 15 May 2011 to 15 June 2011 in five different time slots across 12 TV channels, including the Chongqing Channel, news, and video channels. The advertisement intended to raise public awareness and motivate smokers to quit smoking. The advertisement highlighted severe consequences of exposure to secondhand smoke on colleagues and other non-smokers. The ad sought to discourage ex-smokers from smoking and to promote public awareness of the hazards of smoking, especially, passive smoking in the office. The evaluation intended to assess the reach, recall, and comprehension of campaign messages among the audience and to assess perceived impact of the messages on attitudes and likely future behaviors of people.

### Participants and methods

#### Research method

This study adopted a post-campaign and street-intercept design. A post-campaign evaluation of the “Invisible Killer-Office Campaign” was conducted. The campaign featured a television advertisement adapted from an original 2007 campaign by the UK National Health Service [14]. The campaign message was appraised as intended, as the vast majority of respondents said the ad was easy to understand, personally relevant, provided new information, and made them stop and think.

#### Ethical approval

The study protocol was approved by the Ethics Committee of Chongqing Medical University. All study participants gave informed written consent.

#### Population and sample

A questionnaire survey was administered in June 2011. Data were obtained from four districts (Yuzhong, Shapingba, Jiulongpo, and Nanan), which were randomly selected from nine urban districts of Chongqing in Western China by computer. Individual inclusion criteria included 1) aged 18–45 years at the time of the study, 2) lived in Chongqing City during the time of the study, and 3) residing in Chongqing for over six months prior to the study. In 1342 target interviewers, 19 persons denied answering any questions, and the preliminary response rate was 98.6 % (1323/1342). Among 1323 respondents, 1265 persons answered all questions, giving a final sample of 1265 in the analysis. Research participants consisted of 1,014 (80.16 %) males and 251 (19.84 %) females. According to [15], the prevalence rate of smoking in China is about 28.1 %. We set *P* = 0.3; *Q* = 1 − *P* = 1 − 0.3 = 0.7; margin of error *d* = 0.10*P* = 0.10 × 0.30 = 0.03, *Z*_*α*_ = 1.96. The sampling size is $N=\frac{Z_{\alpha}^2\times pq}{d^2}=\frac{1.96^2\times 0.3\times 0.7}{0.03^2}=897$. The total sample size was 1265 in the survey.

### Sampling framework

The following guidelines were implemented during the street intercept survey.

1. The entire geographical region of Chongqing needed to be accurate in the investigation. Considering all cities and towns in Chongqing, the survey areas were divided into four to six parts according to the geographical vicinity with certain socio-economic characteristics of the communities, which can be set to one.
2. At least one or two selected sites had a gathering of a large number of people in a short time in four to six communities, with respect to different characteristics. Through the above steps, we selected eight to twelve data collection sites from four to six communities. From two data collection sites from every community, we distributed the expected number of respondents.
3. In accordance with the procedure described above, we invited people to participate in the survey in each study site. We randomly selected four districts in Chongqing (Shapingba, South Bank, Yuzhong District, and Jiulongpo District). In each district, we chose two sites, namely the square (Three Gorges Square, Yangjiaping pedestrian street, Jiefangbei pedestrian street, Nanping walking street) and the university.

### Questionnaire

The questionnaire was translated from English to Chinese by a team named Applied Group of Translation whose members are college students, who were under the guidance of professional English teachers. The questionnaire was finalized after repeated discussions with experts and after pilot investigation. We modified the questionnaire according to the results of preliminary investigation, especially on the presentation of questions and further improved the answer options of the questions. The revised questionnaire had an acceptable level of face and content validity and readability. Demographic characteristics, namely, age, educational level, dwelling time (categorized as six months to one year and above one year) were assessed by self-reporting. However, trained investigators facilitated the answering of smoking-related knowledge-attitude-practice and knowledge and attitude about tobacco-related diseases, smoking cessation-related thoughts and practices, and past smoking practices. Education levels were categorized as primary school or below, junior middle school (basic education), senior high school (including vocational/technical secondary school and junior college, and secondary education), and senior college and university (higher education) or above. Age was categorized as 18–25 years, 26–35 years, and 36–45 years. The questionnaire items asked for respondent data on prevalence of cigarette and other tobacco use, initiation, susceptibility, perceptions, attitudes, smoking-related behaviors, exposure to secondhand smoke, anti-smoking TV advertisement, and basic demographic information. Current smokers included all patients smoking tobacco on a daily basis. We asked all respondents whether they had smoked cigarettes in their lifetime. A current smoker was defined as a person who smoked tobacco at the time of the interview. Recognizing the advertisement was defined based on the “yes” answer of respondents to “Recently, there was an ad on television in which we see a man smoking in his office and the black cigarette smoke spreading everywhere. The ad has the following the campaign slogan, ‘second hand smoke, invisible killer.’ Have you seen this ad? [SHOW CARD WITH CAMPAIGN IMAGES].” To avoid interference knowledge of the effects of smoking and passive smoking, we measured if the respondent had seen the advertisement at the end of the questionnaire.

### Effect measure of anti-smoking television advertising

This study measured the effects of an anti-smoking television advertisement. The participants were required to answer the following questions:

(1) Has the ad made you stop and think? (Yes/No); (2) Was the ad relevant to your life and you? (Yes/No); (3) Has the ad provided new information to you? (Yes/No); (4) Has the ad made you concerned about the effects of your smoking on my health? (Yes/No) [Smokers only]; (5) Has the ad made you feel concern about the effects of your smoking on the health of the person around you? (Yes/No) [Smokers only]; (6) Have you said something about the ad or discussed the ad with others? (Yes/No); (7) Have you tried to persuade others to quit smoking? (Yes/No); (8) Have you thought of quitting smoking? (Yes/No) [Smokers only]; (9) Have you looked into ways to quit smoking? (Yes/No) [Smokers only]; (10) Have you made an attempt to quit smoking? (Yes/No) [Smokers only]; (11) Has the ad made you more likely to take steps to reduce your exposure to smoke from other person’s cigarettes? (Yes/No) [Non-smokers only]; (12) Has the ad made you more likely to avoid exposing others to your cigarette smoking? (Yes/No) [Smokers only]; (13) Has the ad made you more likely to quit? (Yes/No) [Smokers only].

### Pilot test

The pilot test was conducted in a medical university. A total of 30 individuals participated in this pretest. We modified the questionnaire according to results of the pilot test, especially on the presentation of questions.

### Survey implementation

During the actual data gathering, participants were selected at each survey site and were kindly approached to indicate their willingness to participate in the campaign. Persons who consented were shown the TV ad, “An Invisible Killer in the Office,” and interviewed face to face by researchers to answer every item in the questionnaire. The interviewers read all the questions in the questionnaire to the respondents and marked answers as appropriate. Each interview lasted from 10 to 20 min.

### Survey administration

The investigation was carried out after an explanation of the purpose of the research by investigators who had undergone standardized training and familiar with the objectives and methodology. Data collection at each site took place at multiple times (minimum twice a day) through the day and two interviewers stood separately at different points of the data collection site to get the most number and type of person. Interviews were conducted at predetermined and consistent times in the mornings, afternoons, and/or late evenings. Data collection spanned at least three to five days.

### Respondent selection and interviewing

To be consistent on the invitation of persons to participate in the survey, interviewers approached every fifth adult who passed their way to participate in the survey. As long as the person looked like an adult, this person was approached. If the person was aged over 45 years old, a method described below was applied to determine eligibility for the survey and to discontinue the interview. Once every fifth person was approached, the interviewer must introduce him/her and the purpose of the research and seek their participation.

- If the person refused to participate, the interviewer thanked the person for his/her time and marked a note of the date, time, and reason for the refusal.
- If the person agreed to participate, the interviewer thanked the respondent, opened a fresh survey packet, and asked the person preliminary “screening” questions to ensure eligibility. This screening section include questions such as the person’s age and place of residence.

### TV ad “An Invisible Killer in the Office [16]”

The advertisement was the same as Smoke-Free Guangzhou 2010, but without the last sentence on TV. “*Secondhand smoking was harmful and neglected*” was the theme of the propaganda, and was broadcasted as public service advertising. This advertisement aimed to teach people the dangers of smoking and smoking around others. “SHS Invisible Killer-Office” was developed from an original ad concept from the United Kingdom and depicted the effects of secondhand smoke on smokers and non-smokers in a typical office environment [17]. Persons can watch the smoke-free advertisement through the mobile phone of the client. The advertisement was also available via street LED to reach people in the community.

### Key message

Secondhand smoke, the invisible killer, attacks the vital organs of everyone.

### Time

The ad was 25 s long with the original Chinese text and speech.

### Details of the advertisement

The scene showed a manager smoking in his office. Everybody is busy working that they failed to notice they are exposed to secondhand smoking.

### Transcript

“Secondhand smoke attacks the vital organs of everyone who breathes it, increasing their chances of heart disease by a quarter, even if they’ve never smoked. The scariest thing is, you can’t even see it; 85 % of secondhand smoke is invisible. Secondhand smoke: the invisible killer.”

### Evaluation highlights

Nearly 1 in 4 (24 %) of the respondents recalled seeing the campaign. The campaign message was appraised as intended. The vast majority of respondents said that the ad was easy to understand, personally relevant, provided new information, and made them stop and think. The ad made both smokers and non-smokers more likely to take steps to reduce children’s exposure to SHS exposure (91 % and 96 %, respectively). Meanwhile, 94 % of non-smokers said that the ad made them more likely to protect themselves from SHS exposure. Over 80 % of smokers and non-smokers agreed that an indoor smoke-free law would benefit public health and clients at indoor places had the right to breathe clean air. Moreover, 83 % of non-smokers and 64 % of smokers felt that indoor smoke-free laws would help smokers to quit [18].

### Advertising schedule of “An Invisible Killer in the Office”

The advertisement entitled “An Invisible Killer in the Office,” was broadcasted daily from 15 May 2011 to 15 June 2011 in five different time slots across 12 TV channels, including the Chongqing Channel, news, and video channels (see Table 1).

**Table 1:** Advertising schedule of “An Invisible Killer in the Office”

Channel	Number of advertising schedule	Schedule1	Schedule2	Schedule3	Schedule 4	Schedule5
CQTV (news)	5	11:28	14:17	16:57	22:28	00:33
CQTV (television)	5	07:36	13:05	17:58	21:16	00:58
CQTV (city)	5	07:55	09:20	17:49	19:49	21:49
CQTV(entertainment)	5	09:24	16:26	19:26	20:56	22:52
CQTV (public)	5	09:35	11:23	14:10	20:00	21:00
CQTV (science and education)	5	09:00	10:30	13:50	17:00	20:10
CQTV (fashion)	5	08:55	17:00	00:14	00:46	02:07
CQTV (life)	5	08:58	15:55	17:53	00:24	01:25
iTV guide channel	5	12:36	17:11	18:06	20:09	21:20
Digital charm fashion channel	5	07:35	11:58	12:50	14:27	19:52
Digital acrobatic channel	5	12:41	15:38	17:38	20:58	23:38
Digital new financial channel	5	10:20	11:58	17:58	20:58	00:58

### Data analysis

The data gathered from the questionnaires were double-checked before data entry using Epi-data3.1 software. Data were meticulously sorted, cleaned, and analyzed using Statistical Analysis System software (version 9.1; SAS Institute, Cary, NC). Incomplete or missing data were excluded and all data entries were double-checked to prevent errors. Descriptive statistics were utilized in data analysis. Statistical tests included a two-sided test and statistical significance was considered at *p* < 0.05. In addition, logistic regression analysis was employed to evaluate the effect of TV advertisement on Chinese audience. Several factors were considered in the modeling of advertisement effect measures, namely, “age,” “gender,” “smoking status,” “attitude toward the regulation prohibiting smoking in public places and the workplace,” “educational level,” and “duration of residency in Chongqing.” Five advertisement effect measures are “The ad made me stop and think (recorded into two categories: 1 = “Yes” and 0 = “No”),” “The ad was relevant to my life and me (recorded into two categories: 1 = “Yes” and 0 = “No”),” “Discuss the ad with others (recorded into two categories: 1 = “Yes” and 0 = “No”),” and “Try to persuade others to quit smoking (recorded into two categories: 1 = “Yes” and 0 = “No”).” The threshold for statistical significance was established at the 0.05 level in the logistic regression.

## Results

### Characteristics of study participants

A total of 1014 (80.2 %) eligible respondents were males. The audience rating of the advertisement entitled “An Invisible Killer in the Office” was 23 %. Significant differences with respect to education level were observed among 238 (18.8 %) individuals who reported seeing the advertisement and among 1027 (81.2 %) participants who said that they did not see the advertisement. More than 90 % of respondents were against smoking in public places. Table 2 shows the demographic characteristics of the research participants.

**Table 2:** Characteristics of the study participants, Chongqing, China, 2011 (*n* = 1,265)

Variables	Seen the advertisement entitled *“An Invisible Killer in the Office”*		Statistical tests
	YES (%)	NO (%)	
Gender			
Male	180 (75.6)	834 (81.2)	*χ* ^2^ = 3.7788, *p* = 0.0519
Female	58 (24.4)	193 (18.8)	
Age			
18–25 years	146 (61.3)	689 (67.1)	*χ* ^2^ = 5.2799, *p* = 0.0714
26–35 years	49 (20.6)	209 (20.4)	
36–45 years	43 (18.1)	129 (12.6)	
Education level			
Basic education	25 (10.5)	64 (6.2)	*χ* ^2^ = 17.4428, *p* = 0.0002
Secondary education	113 (47.5)	384 (37.4)	
Higher education	100 (42.0)	579 (56.4)	
Smoking status			
Smoking	111 (46.6)	425 (41.4)	*χ* ^2^ = 2.1860, *p* = 0.1393
Non-smoking	127 (53.4)	602 (58.6)	
Attitude to the regulation of smoking in public places			
Support the regulation of smoking in public places	14 (5.9)	45 (4.38)	*χ* ^2^ = 0.9786, *p* = 0.3225
Oppose the regulation of smoking in public places	224 (94.1)	982 (95.6)	

Note: Education level was categorized as ≤ primary school, junior middle school (basic education), ≥ senior high school (including vocational/technical, secondary school, and junior college), (secondary education) and ≥ senior college and university (higher education)

### Contact with media and exposure to tobacco control advertisements

In one month immediately preceding the survey, respondents were exposed to advertisements about smoking and health as well as other ads that encouraged quitting on radio (24.0 %), outdoor advertisements or posters (42.9 %), newspapers or magazines/journals (45.4 %), indoor LED screens (48.9 %), mobile TV (55.3 %), and television (65.2 %). However, 69.3 % of respondents claimed not hearing anything about tobacco control advertisements on the radio and 52.2 % reported not seeing any advertisement in newspapers, magazines, and journals about smoking control. Evidently, respondents were more likely to have noticed tobacco control advertisements on television (65.2 %) and mobile TV (55.3 %) than in other media. Moreover, 63.0 % of interviewers claimed receiving messages via their mobile phones every day. (Refer to Table 3.)

**Table 3:** Contact frequency of mass media and anti-tobacco advertising media in Chongqing, China, 2011 (*n* = 1265, %)

Variable	Frequency of media contact within one week					Exposure to smoking control ads from media in the past one month		
	Never	One times a week	Two to three times a week	Four to six times a week	Everyday	YES	NO	Inapplicability
Television	240(19.0)	217(17.2)	318^▲^(25.1)	197(15.5)	293(23.2)	825 (65.2)^▲^	430(34.0)	10(0.8)
Radio	706^**▲**^(55.8)	244(19.2)	175(13.8)	53(4.19)	87(6.9)	304(24.0)	876(69.3)^▲^	85(6.7)
Newspapers	301(23.8)	330(26.1)	333^**▲**^(26.3)	124(9.8)	177(14.0)	574(45.4)	660^**▲**^(52.2)	31(2.5)
Magazines	337^▲^(26.6)	434(34.3)	313(24.7)	95(7.5)	86(6.8)			
Mobile phone messages	137(10.8)	99(7.8)	109(8.6)	123(9.7)	797^▲^ (63.0)	--	--	--
Social Network Site	338(26.7)	157(12.4)	183(14.5)	119(9.4)	468^▲^(37.0)	--	--	--
Mobile TV	--	--	--	--	--	700▲(55.3)	548(43.3)	17(1.3)
Indoor LED screens	--	--	--	--	--	618▲(48.9)	612(48.4)	35(2.8)
Outdoor ads or posters	--	--	--	--	--	543(42.9)	665▲(52.6)	57(4.5)

Note: 1) Mobile TV included bus and subway; Indoor LED screens included hospital; Outdoor advertising included billboards and posters

2) ^▲^Largest number of options

### Effect of advertisement, “An Invisible Killer in the Office,” on urban community population

The advertisement reported that 61.7 % of respondents started to think about quitting smoking. More than 70 % believed that “The ad was relevant to my life and me.” The ad provided new information to 77.5 % of smokers, 77.2 % of non-smokers, and 77.3 % of the total sample. The advertisement resulted in smokers being concerned about the effects of smoking on their health (81.1 %) and on the health of the person around them (80.2 %), such as families, friends, and colleagues. However, only 39.8 % of smokers thought about quitting because of the advertisement. Table 4 summarizes the responses.

**Table 4:** Effect of advertisement, “An Invisible Killer in the Office” on urban community population in Chongqing, China, 2011 (*n* = 238)

Item	Smokers (*n*, %)	Non-smokers (*n*, %)	All respondents
Effect of the advertisement on attitude and practices of all participants			
The ad made me stop and think. [all respondents]	67 (60.4)^▲^	81 (63.8)^▲^	148 (61.7)^▲^
The ad was relevant to my life and me. [all respondents]	85 (74.8) ^▲^	111 (87.4) ^▲^	196 (81.7) ^▲^
The ad provided new information to me. [all respondents]	86 (77.5) ^▲^	98 (77.2) ^▲^	184 (77.3) ^▲^
I say something about the ad or discuss the ad with others. [all respondents]	43 (33.9)	32 (28.8)	75 (31.5)
I try to persuade others to quit smoking. [all respondents]	92 (77.4) ^▲^	33 (29.7)	125 (52.3) ^▲^
Effect of the advertisement on smoker attitude and practices			
The ad made me feel concerned about the effects of my smoking on my health. [smokers only]	90 (81.1) ^▲^		
The ad made me feel concerned about the effects of my smoking on health of the person around me. [smokers only]	89 (80.2) ^▲^		
I think about quitting smoking. [smokers only]	41 (39.8)		
I look into ways to quit smoking. [smokers only]	46 (44.7)		
I make an attempt to quit smoking. [smokers only]	23 (22.8)		
The ad made me more likely to avoid exposing others to my cigarette smoking. [smokers only]	48 (44.0)		
The ad made me more likely to quit. [smokers only]	28 (25.5)		
Effect of the advertisement on non-smoker attitude and practices			
The ad made me more likely to take steps to reduce my exposure to secondhand smoke. [non-smokers only]		83 (72.2)^▲^	

Note: ^▲^More than 50 % of the respondents chose the option

**Table 5:** Comparison of smoking-related knowledge and attitude on the utilization of the advertisement entitled *“An Invisible Killer in the Office”* in Chongqing, China, 2011

Items	Smokers (*n* = 536)		Non-smokers (*n* = 729)	
	Recognized the advertisement	Did not recognize the advertisement	Recognized the advertisement	Did not recognize the advertisement
Smoking cigarettes causes the following:				
Lung disease				
Yes (%)	109 (98.2)	391 (93.1)	127 (98.4)	591 (97.8)
*p*-Value	*p* = 0.041*		*p* = 0.661	
Oral cancer				
Yes (%)	67 (60.4)	233 (55.6)	86 (67.2)	362 (60.3)
*p*-Value	*p* = 0.369		*p* = 0.148	
Heart disease				
Yes (%)	46 (41.4)	167 (39.9)	67 (52.3)	294 (48.8)
*p*-Value	*p* = 0.762		*p* = 0.461	
Stroke				
Yes (%)	21 (18.9)	88 (21.2)	42 (33.3)	183 (30.7)
*p*- Value	*p* = 0.606		*p* = 0.563	
Impotence				
Yes (%)	34 (30.9)	98 (23.6)	44 (34.6)	170 (28.5)
*p*- Value	*p* = 0.114		*p* = 0.166	
Secondhand smoke causes the following:				
Lung cancer in non-smokers				
Yes (%)	99 (89.2)	310 (74.0)	110 (85.3)	484 (80.4)
*p*-Value	*p* = 0.001*		*p* = 0.198	
Lung diseases in children				
Yes (%)	96 (86.5)	311 (74.2)	114 (88.4)	504 (83.7)
*p* Value	*p* = 0.007*		*p* = 0.185	
Heart disease				
Yes (%)	50 (45.0)	160 (38.2)	58 (45.7)	254 (42.3)
*p* -Value	*p* = 0.189		*p* = 0.490	
Birth of low-weight babies				
Yes (%)	80 (72.1)	270 (64.3)	101 (78.3)	434 (72.0)
*p*-Value	*p* = 0.124		*p* = 0.142	
Smoking-related attitude				
Cigarette smoking can cause serious harm to one’s health.				
Agree (%)	92 (82.9)	354 (84.3)	118 (91.5)	539 (89.2)
*p*-Value	*p* = 0.720		*p* = 0.450	
I am concerned about my health when someone is smoking near me.				
Agree (%)	77 (69.4)	282 (67.3)	119 (92.2)	532 (88.2)
*p*-Value	*p* = 0.679		*p* = 0.186	
Exposure to smoke from another person’s cigarette causes heart attack.				
Agree (%)	58 (52.3)	202 (48.2)	90 (69.8)	382 (63.3)
*p*-Value	*p* = 0.449		*p* = 0.167	
[Smokers only] The person around me believes that I should not smoke.				
Agree (%)	80 (74.8)	296 (71.7)		
*p*-Value	*p* = 0.524			
[Smokers only] Quitting smoking would improve my health.				
Agree (%)	84 (79.2)	325 (78.7)		
*p*-Value	*p* = 0.901			

Note: 1) * with statistical difference

2) Birth of low-weight babies: Birth of low-weight babies when the pregnant mother has been exposed to cigarette smoke

3) Persons around me (including family members, friends, and colleagues) believe that I should not smoke

4) Lung disease: lung cancer, respiratory infections, and bronchitis

5) Lung diseases in children: asthma and pneumonia

### Comparison of anti- tobacco TV advertisement effect on smoking-related knowledge between smokers and non-smokers

The perception of the dangers of cigarette use indicated differences between participants who saw the advertisement and those who did not. The breakdown is as follows: smoking cigarettes causes lung diseases (98.3 % vs. 96.0 %), exposing to secondhand smoke causes lung cancer in non-smokers (87.4 % vs. 78.0 %) and in children (87.8 % vs. 79.8 %), smoking causes heart diseases (47.5 % vs. 45.4 %), stroke (27.3 % vs. 27.5 %), and impotence (32.8 % vs. 26.1 %). (Refer to Table 5.).

Significant differences were determined in the mean scores of cognition in “smoking causes lung disease (*p* = 0.041),” “secondhand smoke causes lung cancer in non-smokers (*p* = 0.001),” “secondhand smoke causes lung diseases in children (*p* = 0.007)” between smokers who saw the advertisement and those who did not. However, no statistically significant difference were found on smoking-related knowledge and attitude in items, namely, “smoking causes lung disease,” “secondhand smoke causes lung cancer in non-smokers,” “secondhand smoke causes lung diseases in children.” Moreover, regardless of non-smokers recognizing the ad or not, non-smokers had higher level of knowledge on the effects of smoking than smokers. Differences in knowledge about smoking were found between participants who saw the advertisement and those who did not. (Refer to Table 5.).

### Anti- tobacco TV advertisement effects on smoking-related behaviors

Results showed that 33.9 % of smokers talked about the advertisement or discussed it with others, 77.4 % of smokers attempted to persuade other smokers to quit smoking, and 29.7 % of non-smokers tried to convince other smokers to stop smoking. Furthermore, 22.8 % of smokers attempted to quit smoking, but 60.2 % did not consider quitting. Of the smokers who thought of quitting, 44.7 % searched for approaches to quitting, 44.0 % claimed consciously avoiding exposing others to their cigarette smoking, and 25.5 % believed that the anti-smoking TV advertisement could motivate quitting. (Refer to Table 4.)

### Logistic regression model for identifying factors that affect anti- tobacco TV advertising entitled “An Invisible Killer in the Office”

Several factors were considered in the modeling of the effects of anti-tobacco TV advertising, including age, gender, smoking status, attitude toward the regulation prohibiting smoking in public places and the workplace, educational level, and duration of residency in Chongqing. Logistic regression analysis on socio-demographic data (Table 5) indicated personality and cognitive factors. Females (51.7 %) were less likely to stop and think about smoking than males (65.6 %) (OR = 0.517, 95 % CI [0.281–0.950]) and that non-smokers (87.4 %) were more likely to find the advertisement relevant than smokers (74.8 %) (OR = 2.34, 95 % CI [1.19–4.61]). No significant predictor was found regarding discussing the advertisement with others. Respondents aged 26–35 years (67.4 %) were more likely to persuade others to quit smoking than respondents aged 18–25 years (36.3 %) (OR = 0.457, 95 % CI [0.215 -0.974]). (Refer to Table 6.)

**Table 6:** Logistic regression model to predict favorable attitude towards the four advertisement effect measures in Chongqing, China, 2011 (*n* = 238)

Item	The ad made me stop and think		The ad was relevant to my life and me		I discussed the ad with others		I tried to persuade others to quit smoking	
	Agree	OR(95 % CI)^a^	Agree	OR(95 % CI)^a^	Agree	OR(95 % CI)^a^	Agree	OR(95 % CI)^a^
Gender (*n*, %)								
Male^▼^	118(65.6)	1.00	143(79.4)		125(69.4)		100(55.6)	
Female	30(51.7)	0.517(0.281,0.950)^**^	51(87.9)		38(65.5)		13(22.4)	
Age (*n*, %)								
18-25 year^▼^	85(58.22)		120(82.2)		97(66.44)		53(36.30)	1.00
26-35years	35(71.4)		40(81.6)		32(65.3)		33(67.4)	0.457(0.215,0.974)^**^
36-45years	28(65.1)		34(79.1)		34(79.1)		27(62.8)	0.567(0.257, 1.248)
Education level (*n*, %)					-			
Basic education^▼^	18(72.0)		21(84.0)		16(64.0)		13(52.0)	
Secondary education	72(63.2)		92(80.7)		78(68.4)		59(51.8)	
Higher education	58(59.0)		81(81.8)		69(69.7)		41(41.4)	
Smoking status								
Smokers^▼^	67(60.4)		83(74.8)	1.00	79(71.2)		78(70.3)	1.00
Non-smokers	81(63. 8)		111(87.4)	2.34(1.19,4.61)^**^	84(66.1)		35(27.6)	0.199(0.11,0.36)
Dwelling time								
Six months to one year^▼^	16(51.6)		24(77.4)		19(61.3)		20(64.5)	1.00
Above one year	132(63.8)		170(82.1)		144(69.6)		93(44.9)	2.14(0.88, 5.19)
Attitude to the regulation of forbidding smoking in public places								
Support^▼^	142(63.4)	1.00	184(82.1)		150(67.0)	1.00	102(45.5)	
Oppose	6(42.9)	0.36(0.12, 1.09)	10(71.43)		13(92.9)	6.41(0.82,49.96)	11(78.6)	

Note: 1) ** Statistically significant (*P* < 0.05)

2) Education level was categorized as ≤ primary school, junior middle school (basic education), ≥a senior high school (including vocational/technical, secondary school, and junior college), (secondary education) and ≥ senior college and university (higher education)

3) ^a^Logistic regression model was adjusted for gender, age, education level, smoking status, dwelling time, and attitude to the regulation of forbidding smoking in public places

4) ^▼^Reference group

## Discussion

Only approximately 20 % of respondents recognized the advertisement, “An Invisible Killer in the Office.” However, this result did not fail the research expectations because the percentage may involve millions of persons considering the huge base of urban population in Chongqing. Many instances could have influenced recall rate, with the audience rating of Chongqing TV having the most significant effect on audience acceptance of the advertisement. Of the 12 Chongqing TV channels, most are commercial channels characterized by profit-seeking advertisements, unpopular programs, and low audience rating. MMCs could directly and indirectly produce positive or negative changes in health-related behaviors across large populations [19]. Hence, the collective experience in campaign research and evaluation showed that health education on smoking control could be transmitted by a variety of media, especially by the TV channels with high audience rating and popular media (CCTV, mobile phone, and Internet) to expand public acceptance of the advertisement. Program planners need to examine carefully relative and cost effectiveness of mass media channels they use to promote tobacco control and to identify critical points when shifting limited media resources from television to other media [20].

Tobacco control advertisements were more likely to be noticed on TV and mobile TV than on other media among the urban population. On the one hand, more than half of the respondents reported that they did not see any information about smoking control in newspapers, magazines, outdoor advertising, and journals. This finding indicates that the coverage percentage of a variety of common mass media on publicity of tobacco control knowledge is less than 50 %. Population-based studies have shown that anti-smoking advertisements that used graphic imagery and testimonials to generate high levels of negative emotion are associated with greater recall and influence on smoking attitudes and intentions [21–24]. Therefore, we can continue to use TV to spread information about smoking control.

On the other hand, more than half of the respondents did not hear any smoking control advertisements on the radio. Previous research has found that compared to televised anti-smoking advertisements, radio anti-smoking advertisements could also generate recall, cognitive and emotional responses, and motivation to quit smoking to a similar extent [25]. Thus, prospective tobacco advertising among urban community populations in the future need to consider previously neglected mass media that are easily accessed and often used by people.

Radio can be an effective medium in promoting tobacco control [8]. The increasing number of vehicles in developing countries allows for fully using the car radio to promote smoking control and continual use of trains for travel makes the train radio a good medium for anti-smoking advertising. Thus, adapting new and widespread MMC material and mass media can be an effective and economic strategy to promote tobacco-smoking control.

Despite knowing that “smoking is a health hazard,” urban persons have cognitive limitations on specific knowledge of the dangers of smoking. Most of the research participants know that smoking can cause lung diseases, oral cancer, and heart ailments, but only a few know that smoking can trigger stroke, erectile dysfunction (ED), and other smoking-related illnesses. Therefore, health educators need to add new knowledge and to learn more related research to expand the cognition of the public. This study further confirmed that non-smokers had higher level of knowledge of the hazards of smoking than smokers [26]. Hence, the ad should instead be focused on the smoking population. More than 90 % of respondents opposed smoking in public places. Similarly, data from the ITC China survey showed that over 70 % participants supported a comprehensive smoke-free policy in school, health-related facilities, government buildings, and in taxis [8]. Previous studies have shown that mass media were vital in promoting and reducing tobacco use in the United States [27]. Mass media marketing of tobacco products through direct advertising and product placement in cultural and entertainment events has been related to increased tobacco use [28, 29]. Although majority of respondents supported smoking control policy, the prevalence of secondhand smoke was serious.

This study confirmed that tobacco advertising has a definite effect on the cognition and behavior of urban community population, specifically in countries where TV is a pervasive mass medium, as shown by previous research on the effects of tobacco advertising in the adults [7]. The advertisement, “An Invisible Killer in the Office,” shows that smoking control advertising can prompt a smoker to desire and consider quitting, but has less significant impact on smokers to actually quit smoking. Only a small proportion of smokers have taken active steps to quit smoking and some are prepared to quit after exposure to the advertisement. In addition, smoking control advertisement was likely to improve smoker recognition that smoking is harmful to the lungs of both smokers and passive smokers, and to urge some non-smokers to attempt persuading others to quit and to avoid passive smoking. Moreover, television ads that graphically communicated serious harms of tobacco use were likely to be effective with smokers in low- to middle-income countries [30]. However, this study showed that this ad did not show differences on smoking-related knowledge and attitude between non-smokers who had seen the ad and those who did not. This difference may indicate that non-smokers have higher level of knowledge of the effects of smoking, and thus, the advertisement had no effect on non-smokers. Non-smokers, regardless of whether they recognized the ad, had the same or higher level of knowledge of the effects of smoking than the smokers. Thus, the ad may not be the right form for making those changes and the ad should instead focus on the smoking population.

Although this ad did not show differences on smoking-related knowledge and attitude among non-smokers, we analyzed the study results based on smoking status of the respondents. The comparison showed that advertising had significant effects on smokers, yet non-smokers had higher level of knowledge of the effects of smoking. Therefore, the advertisement had no effect on non-smokers. Moreover, the number, frequency, and duration of ads, without enough doses of ads for non-smokers, may not result in differences in smoking-related knowledge and attitude among non-smokers. Population-wide interventions that can reduce adult smoking prevalence are critical to curb the pandemic of tobacco-related disease [31, 32]. Evidence suggests that anti-smoking media campaigns may be potentially helpful in reducing smoking among those exposed to the message [33, 34]. Many other effects could not have been found in the present research, and thus, continual monitoring is needed for the effect of anti-smoking TV advertising.

The effectiveness of anti-smoking TV advertisements among urban population could be influenced by numerous factors in China. For logistic regression analysis, significant differences are explored. Gender is one such factor that affects attitude toward smoking, as shown by the result that females were less likely to stop and think about quitting than males. Smoking status is likewise an important factor that affected attitude towards “The ad was relevant to my life and me,” as shown by the result that non-smokers were more likely to believe such attitude than smokers. Age was another factor, as shown by the result that respondents aged 26–35 years were less likely to attempt persuading others to quit smoking than respondents aged 18–25 years. However, overall results indicated few differences in responses to the ad by demographics, smoking status, and smoke-free attitudes. This result may be the more important finding, and indicate that the number, frequency, duration, and doses of ads may affect the effectiveness of anti-tobacco advertisement. Therefore, anti-tobacco advertising designers need to consider fully these factors in future planning of TV advertisements.

### Strength and limitations

The study identified factors on demographics of the smoking population who were more likely to respond to a TV anti-smoking advertisement. An anti-tobacco MMC in urban western China on TV advertising for tobacco control was evaluated and factors determining the effects of an anti-smoking TV advertisement on smokers and non-smokers in China were identified. The information on viewership and campaign recall is likely to be useful especially in urban communities as well as less developed areas in developing countries, such as China. Municipal health practitioners and educators may carry out preventive interventions on smoking by teaching smokers about the risks inherent in cigarette smoking to motivate them to attempt to quit.

Notwithstanding the above strengths, this study has certain limitations. First, the study did not control for potential confounding between the campaign-aware and campaign-unaware groups because the small sample size rendered this task relatively difficult to achieve. Second, cross-sectional survey data reduced the ability to make direct causal inferences, to explore whether unmeasured factors may better explain the observed relationships we observed, and to determine the direction of causality. Precise causal inferences required behavior (i.e., smoking status) monitoring after exposure to the advertisement. Third, the face-to-face survey administration design may bring about information bias. Respondents may not have answered the questions truthfully, especially when questions were considered sensitive given the overall tobacco control environment in China. However, all questions in the survey were reviewed by a research panel and participants in the pilot study, and thus, the questionnaire was less likely to include items that could be perceived as sensitive by the study participants. During the face-to-face interview, investigators asked questions one by one to assure that respondents will answer seriously. The investigators were trained prior to conducting the survey the quality of data gathering.

Fourth, the absence of baseline data before the intervention did not allow for a before-and-after comparison and analysis. Respondents shown the TV ad before were not asked the questions about their knowledge of the effects of smoking and passive smoking. Fifth, considering the nature of the delivery of MMCs, the inclusion of individual randomization was not possible in this study, and the possibility that the consequences were biased by unequally distributed antecedent factors affecting outcome cannot be entirely discounted. Sixth, the data were analyzed from one secondhand smoking advertisement launched in one city in western China, and this limitation may affect the study generalizability in other areas. China is the world’s most populous country with 1.3 billion people and more than half of them live in the rural areas [35]. Smoking prevalence was higher among rural residents [36] and particularly, among adult males [37]. In the study, which is a street intercept survey with biased sample, only one in five subjects were females, and this outcome may affect the representativeness of the study population, which requires cautious interpretation of the study results. Last, some factors were not taken into account in this study, such as the time spent on watching the advertisement, level of comprehension, and satisfaction of the advertisement. These factors may be potentially related to advertising effects. Further analysis of these factors will help to understand the effect of advertising. The WHO Framework Convention on Tobacco Control (FCTC) [38] recommended comprehensive tobacco control programs. The incorporation of television-based anti-smoking advertisement into the multiple tobacco prevention and control strategies are expected to combat the grand challenges of chronic diseases [39].

## Conclusion

This study assessed one anti-smoking ad in one Chinese city, and thus, the conclusions may be limited to referring to the effectiveness of this ad in particular. Despite knowing that “smoking is a health hazard,” urban persons have cognitive limitations on specific knowledge of dangers of smoking. This study showed that this ad did not show differences on smoking-related knowledge and attitude between the non-smokers who had seen the ad and those who had not. Thus, this method may not be the right form for making non-smokers change, as the ad should instead be focused on the smoking population. This study found that current anti-smoking TV advertising could affect knowledge and attitudes towards smoking. The overall results indicated few differences in response to the ad by demographics, smoking status, and smoke-free attitudes. Future measures are needed to increase the coverage percentage of common mass media on the publicity of tobacco control knowledge.
